# Exploring the simultaneous σ-hole/π-hole bonding characteristics of a Br⋯π interaction in an ebselen derivative *via* experimental and theoretical electron-density analysis

**DOI:** 10.1107/S2052252518011041

**Published:** 2018-09-01

**Authors:** Rahul Shukla, Nicolas Claiser, Mohamed Souhassou, Claude Lecomte, Shah Jaimin Balkrishna, Sangit Kumar, Deepak Chopra

**Affiliations:** a Department of Chemistry, Indian Institute of Science Education and Research (IISER) Bhopal, Bhopal By-Pass Road, Bhauri, Bhopal, Madhya Pradesh 462066, India; b Cristallographie, Résonance Magnétique et Modélisations, CRM2, UMR 7036, Institut Jean Barriol, CNRS and Université de Lorraine, BP 239, Vandoeuvre-les-Nancy CEDEX F54506, France

**Keywords:** σ-hole bonding, π-hole bonding, ebselen, electron density, molecular electrostatic potentials, crystal engineering, charge, spin and momentum densities, computational modelling, molecular crystals

## Abstract

In this study, the nature and characteristics of a short Br⋯π interaction observed in an ebselen derivative, 2-(2-bromophenyl)benzo[*d*][1,2]selenazol-3(2*H*)-one, has been explored. The electronic nature of this Br⋯π interaction was investigated *via* high-resolution X-ray diffraction and periodic density functional theory calculations using atoms-in-molecules (AIM) analysis. This study unravels the simultaneous presence of σ-hole and π-hole bonding characteristics in the same interaction. The dual characteristics of this unique Br⋯π interaction are further established *via* molecular electrostatic potentials (MESPs) and natural bond orbitals (NBOs).

## Abstract 2

Experimental and theoretical multipolar charge-density analyses of a unique short Br⋯π interaction in an ebselen derivative have been performed. They establish unequivocally the concomitant existence of the characteristics of σ-hole and π-hole bonding in the same interaction.

## 1. Introduction   

Halogen–π interactions (Montoro *et al.*, 2015 ▸; Wang *et al.*, 2016 ▸) represent an important class of interaction due to their significant role in crystal engineering (Reddy *et al.*, 1996 ▸; Hay & Custelcean, 2009 ▸), drug design (Matter *et al.*, 2012 ▸), mol­ecular recognition (Shah *et al.*, 2017 ▸) and protein–ligand interactions (Imai *et al.*, 2008 ▸). The most important aspect of a halogen–π interaction is that it can be classified into two different categories of interaction. Initially, it lies in the category of a σ-hole interaction (Politzer *et al.*, 2013 ▸; Clark *et al.*, 2007 ▸; Murray *et al.*, 2009 ▸), wherein the region of low electron density (σ-hole), localized close to the halogen atom and often characterized by the presence of a positive electro­static potential (Politzer & Murray, 2017 ▸), interacts with the electron-rich region of a π-system, resulting in the formation of a halogen bond (Cavallo *et al.*, 2016 ▸). The second category is the π-hole interaction (Bauzá *et al.*, 2015 ▸), where the lone pair (l.p.) electrons present on the halogen atom can interact with the electron-deficient region of the π-system (π-hole), giving rise to the formation of an l.p.⋯π interaction (Mooibroek *et al.*, 2008 ▸; Egli & Sarkhel, 2007 ▸).

**Figure 1:**
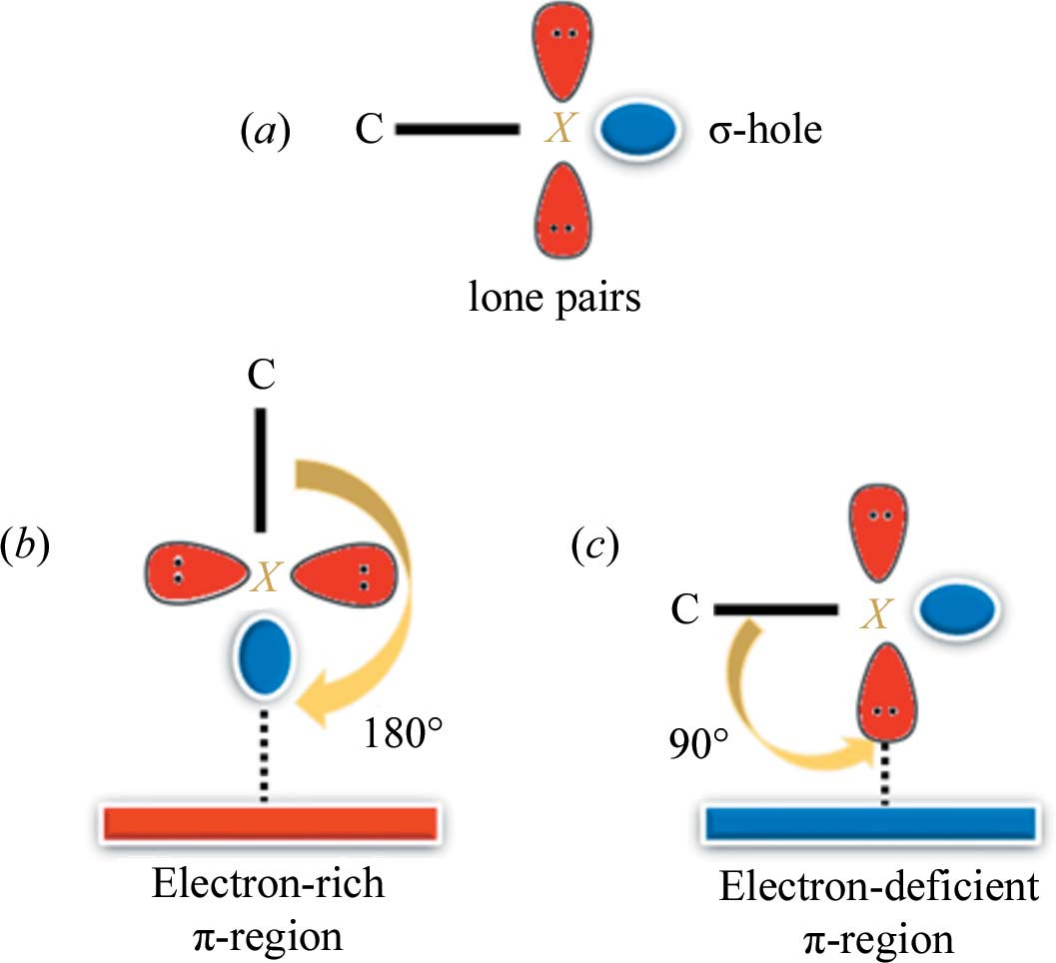
(*a*) A representation of a σ-hole on a halogen atom (*X* = F, Cl, Br, I). (*b*) The ideal geometry for a σ-hole interaction. (*c*) The ideal geometry for a π-hole interaction.

Given that both halogens and π-systems have the capability of acting as both electron acceptor and electron donor, depending on the electronic environment during the formation of the halogen–π interaction, geometric parameters are the primary indicators of the interaction category being a σ-hole or π-hole interaction. Since σ-holes are present along the covalent bonds (Fig. 1 ▸ *a*), the C—*X*⋯π interaction will prefer a linear geometry towards the formation of a σ-hole interaction (Fig. 1 ▸ *b*). The l.p.s on halogens are located nearly perpendicular to the C—*X* bond (Fig. 1 ▸ *a*), so an ideal case of a π-hole directed halogen–π interaction will have a directionality of 90° (Fig. 1 ▸ *c*). While in most cases it is relatively easy to designate the nature of the interaction based on the geometric parameters, an in-depth investigation is needed for the halogen–π interactions wherein the observed directionality has an intermittent value (in the current case the value is 142° and lies between the two extremes). While there are studies investigating the interplay of σ-hole or π-hole interactions (Zhuo *et al.*, 2014 ▸; Politzer & Murray, 2018 ▸; Pal *et al.*, 2015 ▸), experimental validation of the electronic features associated with the dual characteristics of the halogen–π interaction has not been reported in detail to the best of our knowledge. The dual nature of halogens in halogen bonds (as both bond acceptor and bond donor) has been well established since the experimental charge-density analysis of hexachlorobenzene (Bui *et al.*, 2009 ▸) and our paper describes the first experimental charge density of a halogen–π interaction where the simultaneous observations of σ-hole and π-hole bonding are associated with the same interaction.

**Figure 2:**
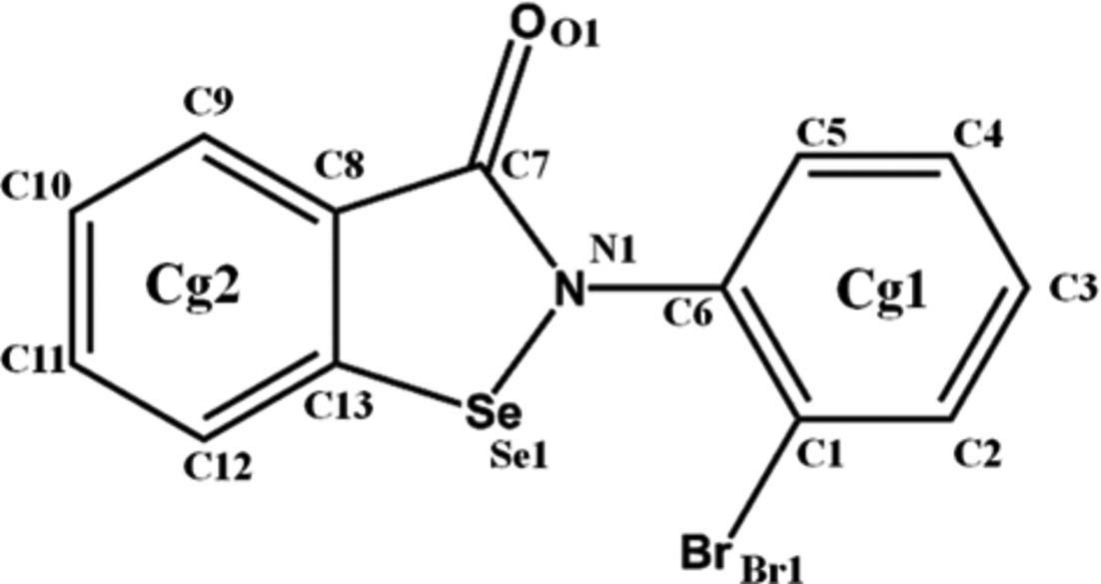
The chemical structure of 2-(2-bromophenyl)benzol[*d*][1,2]selenazol-3(2*H*)-one, α-Se, showing the atom- and ring-numbering schemes.

In this study, we performed an experimental charge-density analysis of the C—Br⋯π interaction present in an ebselen derivative, namely 2-(2-bromophenyl)benzol[*d*][1,2]selenazol-3(2*H*)-one (α-Se) (Fig. 2 ▸), in order to establish the simultaneous presence of σ-hole and π-hole interactions. The experimental results are in line with different theoretical calculations, confirming the dual character of the interaction. Given the importance of both halogen–π interactions (Shah *et al.*, 2017 ▸; Imai *et al.*, 2008 ▸) and ebselen derivatives (Lieberman *et al.*, 2014 ▸; Mugesh *et al.*, 2001 ▸; Zade *et al.*, 2004 ▸; Balkrishna *et al.*, 2014 ▸) in biological systems, this study also provides a new perspective on the concept of bond donor and bond acceptor during the formation of an intermolecular interaction.

## 2. Experimental and computational methods   

### 2.1. Synthesis and crystallization   

2-(2-Bromophenyl)benzo[*d*][1,2]selenazol-3(2*H*)-one (α-Se) was synthesized by the previously reported method (Balkrishna *et al.*, 2010 ▸). The pure compound was crystallized from a saturated solution in dichloromethane:hexane (1:1) at 4°C. Good quality crystals of block morphology were obtained, and one of them was used for the charge-density experiment.

### 2.2. Data collection and details of structure refinement   

The X-ray data for α-Se were collected using Ag *K*α radiation (λ = 0.56086 Å) on a Bruker D8 Venture equipped with a CMOS Photon100 detector at 100 (2) K (Oxford Cryosystem N_2_ cooling system). The data were collected using ω scans with a width of 0.5° per frame up to a resolution of (sinθ/λ)_max_ = 1.123 Å^−1^ with a completeness of 97%. Cell refinement, data integration and data reduction were carried out using the *APEX3* (Bruker, 2015 ▸) software package. Face indexing was performed for numerical absorption correction (Busing & Levy, 1957 ▸; Coppens *et al.*, 1965 ▸). The *SORTAV* (Blessing, 1997 ▸) program present in the *WinGX* (Farrugia, 2012 ▸) software package was utilized for sorting, scaling and merging of the data. The crystal structure was solved by direct methods (Harker & Kasper, 1948 ▸; Karle & Hauptman, 1950 ▸) and first refined on the basis of a spherical-atom approximation based on *F* ^2^ using *SHELXL2014* (Sheldrick, 2008 ▸, 2014 ▸).

### 2.3. Multipole modelling   

The multipolar charge-density refinement was performed against *F* ^2^ using the Hansen–Coppens multipolar model (Hansen & Coppens, 1978 ▸) implemented in the *MoPro/MoProviewer* software package (Jelsch *et al.*, 2005 ▸; Guillot *et al.*, 2014 ▸). The refinement was performed up to a resolution of (sinθ/λ)_max_ = 1.08 Å^−1^ for 9660 reflections with *I* > 3σ(*I*). In the first step of the refinement, the scale factor was refined against all these diffraction data using the *SHELX* refined parameters. Reflections 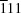 and 012 were affected by the beam stop and had unrealistically large differences between *F*_obs_ and *F*_calc_ which resulted in high residual density, so they were removed from the final refinement. Reflections 002 and 101 were also slightly affected by the beam stop, causing a skewed *F*_obs_/*F*_calc_ ratio at sinθ/λ < 0.1 Å^−1^ (Fig. S1*a* in the supporting information). However, these two reflections do not affect the overall multipolar model and were kept in the refinement process.

In the next step, the position and anisotropic displacement parameters (ADPs) for all the non-hydrogen atoms were refined up to sinθ/λ > 0.7 Å^−1^. Then *P*_val_ (monopole population), *P*_lm_ (multipole population), κ and κ′ (contraction–expansion parameters) were refined in a stepwise manner with all 9660 reflections. *P*_val_ and *P*_lm_ were also refined for the hydrogen atom, for which κ and κ′ were fixed to 1.2. Also, a single κ and κ′ set of parameters was used for the chemically equivalent carbon atoms (a total of five sets of values) present in the molecule.

The C—H distances were constrained to the values obtained from neutron diffraction experiments (Allen & Bruno, 2010 ▸). The ADP values for the hydrogen atoms were estimated using the *SHADE3* server (Madsen, 2006 ▸; Munshi *et al.*, 2008 ▸) and were kept constant throughout the refinement. The multipolar expansion was truncated up to the hexadecapole level (*l*_max_ = 4) for the Se and Br atoms, up to the octapole level (*l*_max_ = 3) for the O, N and C atoms, and up to the dipole level for H atoms. The (*n*_l_, ζ) parameters of the Slater-type radial functions for Se are (4, 8.8), (4, 8.8), (4, 8.8), (4, 8.8) for *l* = 1, 2, 3, 4, respectively. For the Br atom, the (*n*_l_, ζ) parameters of the Slater-type radial functions are (5, 9.732), (5, 9.732), (5, 9.732), (5, 9.732) for *l* = 1, 2, 3, 4, respectively. The values were chosen after trying different radial functions for the Se and Br atoms during the refinement. For the remaining atoms, the default value given in *MoPro* was utilized.

An isotropic extinction correction was applied during the refinement and this significantly improved the residual density around the Se and Br atoms. The topological analysis of the electron density was also performed and visualized using *MoPro/MoProViewer*.

More information concerning the data reduction and multipolar modelling are given in Table 1 ▸. To judge the quality of the modelled electron density, the variations in |*F*_obs_|/|*F*_calc_| with sinθ/λ and of *F*_obs_ with *F*_calc_ are presented in Fig. S1. These maps confirm the very good quality of the diffraction data, despite a few very low- and very high-resolution data.

### 2.4. Computational details   

#### 2.4.1. Theoretical modelling   

Single-point periodic quantum mechanical calculations were performed with the TZVP (Schäfer *et al.*, 1992 ▸; Peintinger *et al.*, 2013 ▸) basis set using the *CRYSTAL09* (Dovesi *et al.*, 2009 ▸) package. The positional parameters obtained from the experimental charge density were utilized for the calculations. The shrinking factors (IS1, IS2 and IS3) and the reciprocal-lattice vectors were set to 4 (with 30 *k*-points in the irreducible Brillouin zone). The bielectronic Coulomb and exchange series values for the truncation parameter were set as ITOL1_ITOL4 = 8 and ITOL5 = 17, respectively, for the calculations. The level shifter was set to 0.7 Hartree per cycle. An SCF convergence limit of the order of 10^−7^ Hartree was used. From the calculation, a total of 12 444 reflections were obtained up to (sinθ/λ)_max_ = 1.08 Å^−1^. For the theoretical charge-density refinement, the ADPs for all atoms were set to zero. During the refinement, the structure factor was assigned unit weight. Multipolar refinement of the theoretical data was carried out up to the same levels as those used for the experimental charge-density refinement. The final *R*(*F*) and *R*(*F* ^2^) were 0.003 and 0.005, respectively, for the theoretical model.

#### 2.4.2. Electrostatic potential maps   

Experimental and theoretical three-dimensional molecular electrostatic potential maps (MESPs) of α-Se were plotted on a Hirshfeld isosurface using the *CrystalExplorer17* software (Turner *et al.*, 2017 ▸) at the MP2/6-311G** level, and also using the *Mopro* viewer (Guillot *et al.*, 2014 ▸).

#### 2.4.3. Natural bond orbital analysis   

The natural bond orbital analysis (Reed *et al.*, 1986 ▸, 1988 ▸) was performed at the B3LYP/6-311G** level using the *NBO6.0* (Glendening *et al.*, 2013 ▸) package integrated with *GAUSSIAN09* (Frisch, 2009 ▸). The *ChemCraft* visualization software (http://www.chemcraftprog.com) was utilized for plotting the bond orbitals between interacting atoms.

## 3. Results and discussion   

### 3.1. Crystal packing   

**Figure 3:**
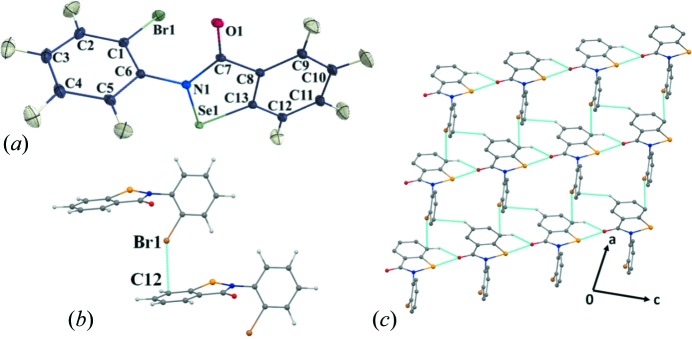
(*a*) An *ORTEP* view of α-Se, drawn with 50% probability displacement ellipsoids at 100 K. Ring *Cg*1: C1–C6; ring *Cg*2: C8–C13. (*b*) The molecular pair formed by the Br⋯π interaction. (*c*) A view of the molecular packing, down the *ac* plane.

The compound α-Se crystallizes in the *P*2_1_/*n* space group with *Z* = 4 (Fig. 3 ▸ *a* and Table 1 ▸). It is interesting to note that the two *Cg*1 and *Cg*2 rings present in the molecule (Fig. 2 ▸) are almost perpendicular to each other, with an angle between their planes of 83.8° (Fig. S7*a*). No other ebselen derivative present in the Cambridge Structural Database (CSD, Version 5.39; Groom *et al.*, 2016 ▸) has such a pronounced deviation from planarity (Fig. S7).

The salient feature in the crystal packing of α-Se is the presence of a short and directional Se⋯O chalcogen bond (Se⋯O = 2.667 Å, N—Se⋯O = 174°) and a C—H⋯O hydrogen bond (H⋯O = 2.37 Å, C—H⋯O = 122°) which form a molecular chain (Fig. 3 ▸ *c*). In a previous charge-density study of ebselen derivatives (Thomas *et al.*, 2015 ▸), the Se⋯O chalcogen-bond-directed dimer was observed to be acting as a supramolecular synthon in the crystal. In their study, the Se⋯O distance ranged from 2.522 to 2.852 Å, while the N—Se⋯O angle ranged from 169 to 175°. Hence, the Se⋯O interaction observed in our study is among the shortest and most directional chalcogen bonds observed in this class of compound.

The molecular chain formed by the chalcogen bond is connected to another similar chain along the *a* axis (Fig. 3 ▸ *c*) *via* a Br⋯π interaction involving the *Cg*2 ring (Fig. 3 ▸ *b*) and utilizes the translation operation (−1 + *x*, *y*, *z*). The Br atom is in close proximity to atom C12 (part of the π-ring), having an intermolecular distance of 3.3042 (8) Å which is ∼0.25 Å less than the sum of the van der Waals radii (Bondi, 1964 ▸). The C1—Br1⋯C12(π) angle of 142.26 (3)° shows that it has an intermediate geometry between that of an ideal σ-hole and a π-hole-directed halogen–π interaction. A search of the CSD for similar C—Br⋯π(C) interactions reveals only one interaction having a Br⋯C(π) distance shorter than 3.30 Å in the angularity range of 120–150° (Table S4). In addition to this, there are 22 unique C—Br⋯π interactions having 120° < C—Br⋯π(C) < 150°, which could also potentially depict simultaneous σ-hole/π-hole bonding characteristics (Table S4), indicating that this type of bonding may be fairly prevalent in structures containing halogens and aromatic groups. Apart from this, the molecular packing of α-Se is also strengthened by the presence of centrosymmetric C—H⋯O=C inter­actions (Table S1).

### 3.2. Multipole refinement   

The electronic features of the crystal structure of α-Se have been explored quantitatively *via* inputs from experimental electron-density analyses performed on crystals of α-Se and based on high-resolution X-ray data (*d* = 0.45 Å) at 100 K, which were later compared with the multipole model obtained from theoretically generated entities. The good quality of the multipole model after the final cycle of refinement was validated by applying the Hirshfeld rigid-bond test to all the covalent bonds involving non-hydrogen atoms.

**Figure 4:**
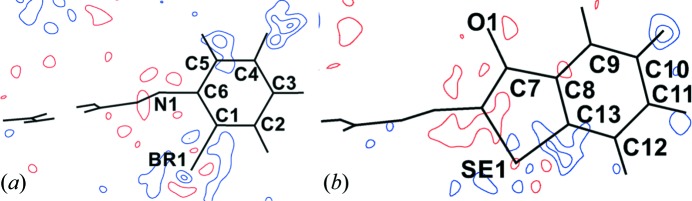
Residual density plots after multipolar refinement, drawn at the 0.1 e Å^−3^ contour level. Positive values are in blue and negative ones in red. Plotted using 9660 reflections [*I* > 3σ(*I*)].

The largest difference in mean-square displacement was observed for the Se1—C13 single bond, 8 × 10^−4^ Å^2^. The fractional dimensional plots were symmetric and parabolic in nature for both the experimental data (Fig. S2) and the theoretical model (Fig. S4). The minimum and maximum residual densities were calculated to be −0.23 and 0.31 e Å^−3^, respectively, for a resolution up to 0.8 Å^−1^, for the experimental model (Table 1 ▸, Fig. 4 ▸). The corresponding values for the theoretical model in the same plane were calculated to be −0.15 and 0.15 e Å^−3^. The residual density is very clean around the Br and Se heavy atoms. The final values of *R*(*F* ^2^) and *wR*_2_(*F* ^2^) for the experimental model were calculated to be 0.0162 and 0.0343, respectively. The final *R*(*F*) and *R*(*F* ^2^) were 0.003 and 0.005, respectively, for the theoretical model, thus confirming the good quality of the multipolar model and of the core and valence wavefunctions used.

The static deformation density and Laplacian maps for both the experimental model (Fig. S3) and the theoretical model (Fig. S5) show essential chemical features such as the anisotropic electron-density distribution around the Br atom. Furthermore, in accordance with previous reports of related experimental charge-density studies (Pavan *et al.*, 2015 ▸), the presence of a charge-depleted region on the Br atom along the extension of the C—Br bond was clearly evident.

### 3.3. Topological analysis   

**Figure 5:**
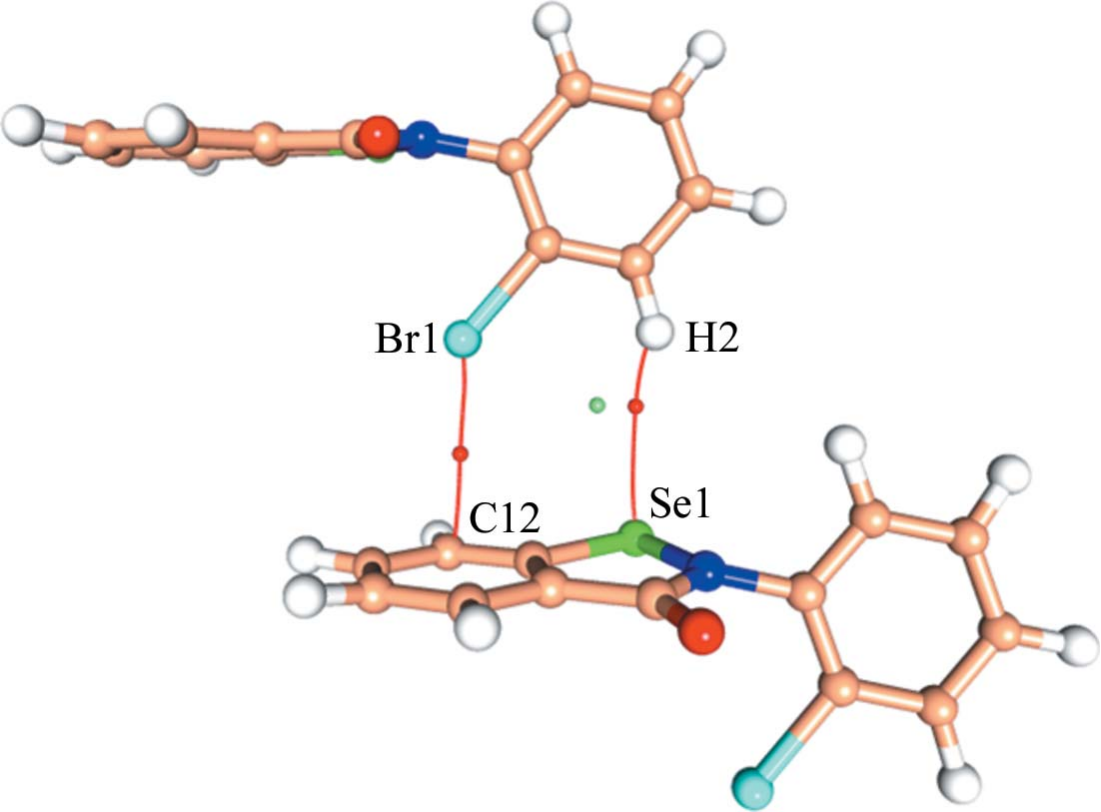
Experimental (3, −1) b.c.p. for Br1⋯C12 and H2⋯Se1 (blue points), and the (3, +1) ring critical point (green).

The multipole models, from both experiment and theory, were used to obtain the topological parameters for the covalent and non-covalent bonds in the solid state. The magnitudes of the ρ and ∇^2^ρ values obtained from the experimental model (Table S2) for covalent Se—C (1.05 e Å^−3^ and −0.30 e Å^−5^, respectively), Se—N (0.99 e Å^−3^ and 4.86 e Å^−5^) and C—N (2.26 e Å^−3^ and −27.50 e Å^−5^) bonds are comparable with the previously reported values for ebselen derivatives (Thomas *et al.*, 2015 ▸). The presence of the Br⋯π interaction is also confirmed by the presence of a (3, −1) bond critical point (b.c.p.) between Br1 and C12 with *R_ij_* = 3.3764 Å (Fig. 5 ▸). In addition to this, a (3, −1) b.c.p. was also observed for the dimer between H2 and Se1 (Fig. 5 ▸). However, the topological parameters at the b.c.p. obtained for Br⋯π are much larger than those observed for the H⋯Se b.c.p. in both the experimental and theoretical models, indicating the dominant nature of the Br⋯π interaction (Table 2 ▸). The higher magnitude of *R_ij_* compared with the Br⋯C bond length shows that the electron density between the interacting atoms follows a curved path. The calculated interaction energy of this mol­ecular pair is −6.5 kJ mol^−1^ from the *PIXEL* method (Gavezzotti, 2011 ▸), which further establishes the stabilizing role of this interaction. For other interactions also, the magnitudes of the topological parameters from the experimental and theoretical models were observed to be similar (Table S3). Topological analysis of the Se⋯O chalcogen bond revealed that the magnitudes of the topological parameters at the (3, −1) b.c.p. (ρ = 0.18 e Å^−3^, ∇^2^ρ = 2.14 e Å^−5^; Table S3) are significantly higher than those observed for the Br⋯π interaction. This shows that the chalcogen bond has a more prominent role than the Br⋯π interaction in the crystal structure of α-Se.

**Figure 6:**
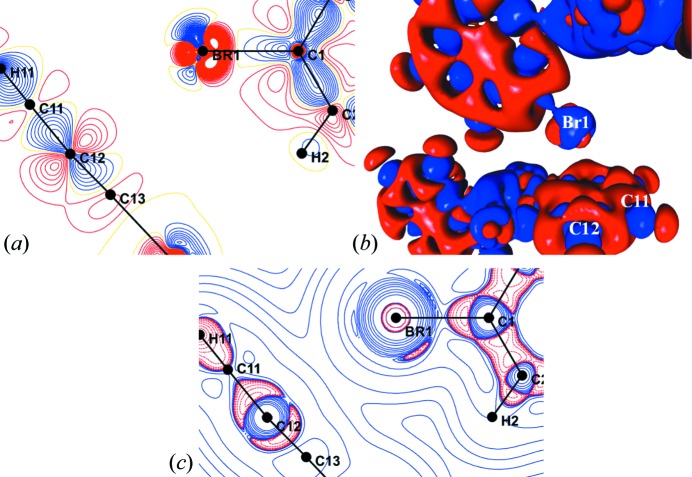
(*a*) Two-dimensional and (*b*) three-dimensional experimental deformation density plots around the bonding region at the ±0.05 e Å^−3^ level. Red denotes charge depletion (VSCD) and blue denotes charge concentration (VSCC). (*c*) An experimental two-dimensional Laplacian drawn on a logarithmic scale (contours in e Å^−5^).

The dual character of the Br⋯π interaction is clearly shown by the two- and three-dimensional experimental deformation density maps (Figs. 6 ▸ *a* and 6 ▸ *b*). The valence-shell charge concentration (VSCC) region (in blue) on the Br atom points towards the charge-depleted region (VSCD, in red) around atom C12, indicating the presence of π-hole bonding characteristics. Also, the charge-concentrated region on the C12—C11 bond of the phenyl ring is appropriately orientated towards the charge-depleted (σ-hole) region of the Br atom, establishing the presence of σ-hole bonding characteristics (Figs. 6 ▸ *a* and 6 ▸ *b*). This dual nature of the Br⋯π interaction is further confirmed by the two-dimensional Laplacian plot, where the VSCC and VSCD regions present on both the halogen bond and the π-bond involved in the interaction are suitably oriented to facilitate the formation of this unique interaction (Fig. 6 ▸ *c*).

### 3.4. Electrostatic potential maps   

**Figure 7:**
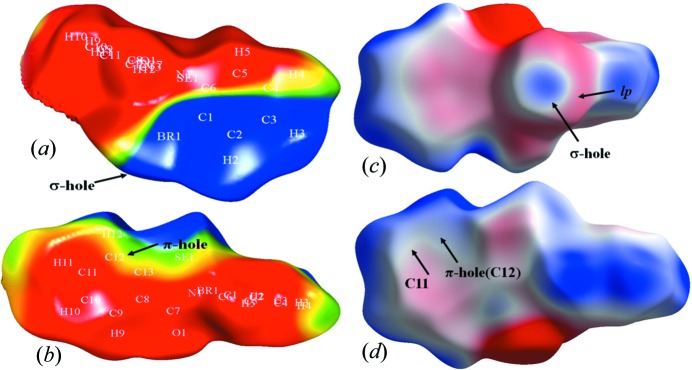
Experimental and theoretical MESPs drawn at ±0.05 a.u., with blue corresponding to positive electrostatic regions and red to negative electrostatic regions. (*a*), (*c*) The σ-hole and l.p. of the Br atom. (*b*), (*d*) The π-hole and electron-rich region along the C11—C12 bond.

Three-dimensional molecular electrostatic potential maps (MESPs) of α-Se plotted using the experimental density (Figs. 7 ▸ *a* and 7 ▸ *b*) and on a Hirshfeld isosurface using the experimental density with *CrystalExplorer17* (Figs. 7 ▸ *c* and 7 ▸ *d*) corroborate the observations made from the electron-density analyses. The σ-hole present on the Br atom along the C—Br bond is clearly evident (in blue), along with the surrounding negative electrostatic region due to the presence of Br lone pairs (in red) (Figs. 7 ▸ *a* and 7 ▸ *c*). This σ-hole is oriented towards the negative electrostatic region present on atom C11, indicating the σ-hole bonding characteristics. On the other hand, the l.p. of Br is oriented towards atom C12, which has a positive electrostatic character (Figs. 7 ▸ *b* and 7 ▸ *d*), hence confirming the π-hole bonding characteristics.

### 3.5. Natural bond orbital analysis   

**Figure 8:**
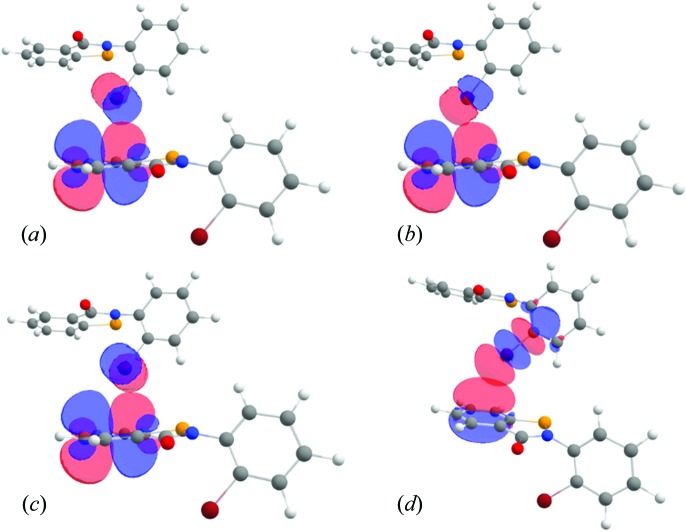
(*a*), (*b*), (*c*) Inter-orbital interactions between three different lone pairs of Br with π*(C—C). (*d*) The inter-orbital interaction between π(C—C) and σ*(Br—C). Blue and red depict positive and negative lobes, respectively.

The observation made from the experimental electron-density analysis and MESP analysis is further confirmed by the natural bond orbital (NBO) analysis performed on the dimer comprising the interactions of interest. This theoretical analysis clearly establishes the occurrence of two different types of interorbital interaction (Table 3 ▸, Fig. 8 ▸). Firstly, corresponding to the π-hole bonding, there is an occurrence of charge transfer from the three lone pairs of the Br atom, *i.e.* l.p.(1), l.p.(2) and l.p.(3), to the π*(C12—C11) orbital, with the second-order perturbation energies *E*(2) corresponding to the transfer being 0.38, 1.09 and 0.79 kJ mol^−1^, respectively. Thus, the total magnitude of *E*(2) for the Br(l.p.) → π*(C11—C12) inter-orbital interaction stands at 2.26 kJ mol^−1^. In comparison, the *E*(2) value corresponding to the π(C11—C12) → σ*(Br1—C1) inter-orbital interaction was calculated to be 2.92 kJ mol^−1,^ which is due to the presence of σ-hole bonding. Hence, the NBO analysis quantitatively supports the simultaneous presence of σ-hole and π-hole bonding characteristics in a single Br⋯π interaction.

## 4. Conclusions   

In conclusion, we have established for the first time experimental evidence for the simultaneous presence of σ-hole and π-hole bonding characteristics in a halogen–π interaction. This study shows that the classification of non-covalent interactions into different categories, such as σ-hole or π-hole interactions, cannot always be done in an absolute manner, as there is always the possibility that the two different regions of the atom participating in the formation of a non-covalent interaction are simultaneously acting as bond acceptor and bond donor.

## Supplementary Material

Crystal structure: contains datablock(s) I. DOI: 10.1107/S2052252518011041/lq5015sup1.cif

Structure factors: contains datablock(s) I. DOI: 10.1107/S2052252518011041/lq5015Isup2.hkl

Supporting information file. DOI: 10.1107/S2052252518011041/lq5015sup3.pdf

CCDC reference: 1816272

## Figures and Tables

**Table 1:** Crystallographic information

Compound name	2-(2-Bromophenyl)­benzo[*d*][1,2]selenazol-3(2*H*)-one
Compound composition	C_13_H_8_Br_1_N_1_O_1_Se_1_
CSD refcode	1816272
Formula weight	353.063
Crystal system	Monoclinic
Space group	*P*2_1_/*n*
*T* (K)	100 (2)
*a* (Å)	7.6043 (7)
*b* (Å)	13.4161 (13)
*c* (Å)	11.8847 (11)
α (°)	90
β (°)	103.676 (3)
γ (°)	90
Volume (Å^3^), *Z*	1178.10 (19), 4
ρ_calc_ (g cm^−3^)	1.991
*F*(000)	680
λ (Å) (Ag *K*α), μ (mm^−1^)	0.56086, 3.498
*T*_min_, *T*_max_	0.275, 0.390
Crystal size	0.15 × 0.24 × 0.39
(sinθ/λ)_max_ (Å^−1^)	1.123
Total No. of reflections	323211
Unique reflections	13583
Redundancy, completeness (%)	22, 97%
*R*_int_ (all)	0.0414
Spherical atom refinement (*SHELX*)	
*N*_ref_ [*I* > 3σ(*I*)]	9660
*R*_obs_	0.0207
*wR*_2_(*F* ^2^)	0.0475
Goodness-of-fit	1.082
Δρ_min_, Δρ_max_ (e Å^−3^)	−1.25, 0.61
Multipole refinement (*MoPro*)	
(sinθ/λ)_max_ (Å^−1^)	1.08
Reflections used [*I* > 3σ(*I*)]	9660
Goodness-of-fit	1.013
*R*(*F* ^2^), *wR*_2_(*F* ^2^)	0.0162, 0.0343
Δρ_min_, Δρ_max_ (e Å^−3^)	−0.23, 0.31

**Table 2:** Topological parameters at the Br⋯C(π) and H⋯Se b.c.p.s in α-Se

	*R_ij_* (Å)	ρ (e Å^−3^)	∇^2^ρ (e Å^−5^)	|*V*|/*G*
Br⋯C(π)				
Experiment	3.376	0.06	0.65	0.80
Theory	3.354	0.08	0.77	0.86
H⋯Se				
Experiment	3.366	0.02	0.25	0.60
Theory	3.331	0.03	0.29	0.71

**Table 3:** Second-order perturbation energy *E*(2) for the Br⋯π interaction

Orbitals involved	*E*(2) (kJ mol^−1^)
Br(l.p.1) → π*(C11—C12)	0.38
Br(l.p.2) → π*(C11—C12)	1.09
Br(l.p.3) → π*(C11—C12)	0.79
π(C11—C12) → σ*(Br1—C1)	2.92
