# Supplementary material for: Exploring the simultaneous σ-hole/π-hole bonding characteristics of a Br⋯π interaction in an ebselen derivative via experimental and theoretical electron-density analysis
Source: IUCrJ. 2018 Sep 1;5(Pt 5):647–53. doi: 10.1107/S2052252518011041 (PMC6126650; doi:10.1107/S2052252518011041)
Supplement: Supplementary file 3 [file m-05-00647-sup3.pdf]

IUCrJ

Volume 5 (2018)

Supporting information for article:

Exploring the simultaneous σ-hole/π-hole bonding characteristics of a Br⋯π interaction in an ebselen derivative *via* experimental and theoretical electron-density analysis

Rahul Shukla, Nicolas Claiser, Mohamed Souhassou, Claude Lecomte, Shah Jaimin Balkrishna, Sangit Kumar and Deepak Chopra

Supporting information

Table of contents

S1. Quality of Multipole modeling
S2. Intermolecular Interactions
S3. Topological Analysis
S4. CSD Analysis

S1. Quality of Multipole modeling

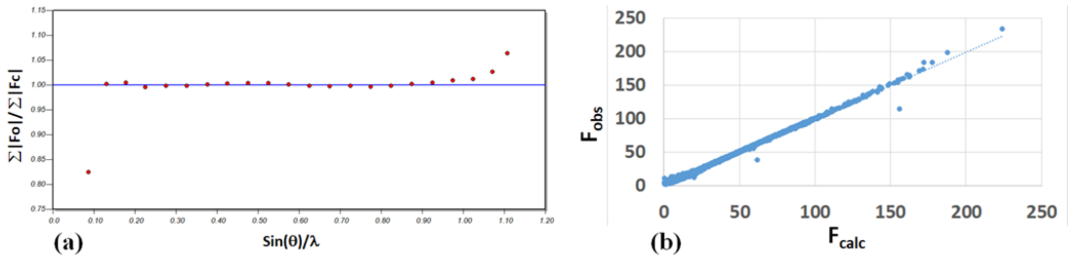

Figure S1 (a) Variation of $|F_{obs}|/|F_{calc}|$ with sin (θ)/λ in **α-Se.** (b) Variation of F_{obs} with F_{calc} in **α-Se**

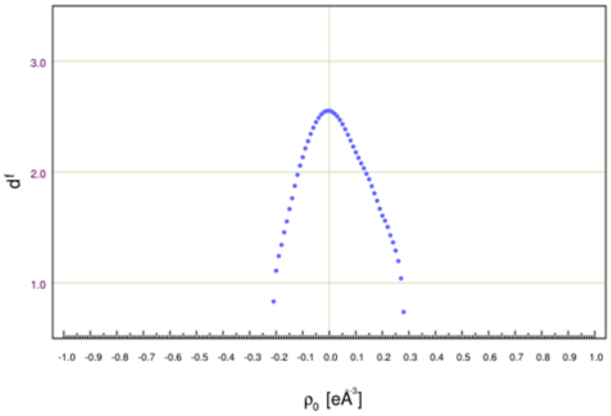

Figure S2 The fractional dimensional plot of an experimental model of **α-Se.**

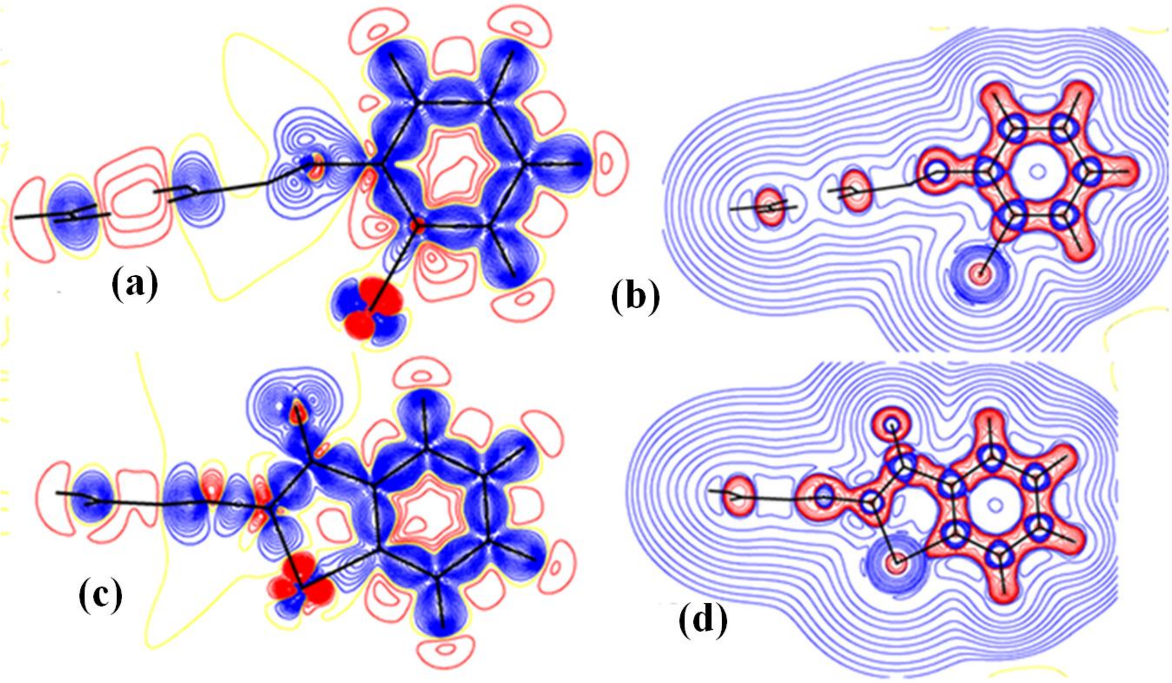

Figure S3 (a), (b): 2D deformation density plots after multipolar refinement drew at 0.05 e/Å^3 contour level. (c), (d) 2D Laplacian plots after multipolar refinement drew at a logarithmic scale. For all, positives values in blue, negatives in red.

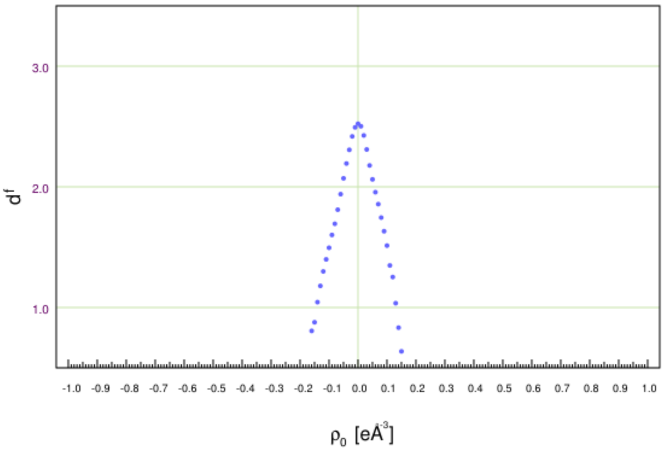

Figure S4 The fractional dimensional plot of a theoretical model of **α-Se.**

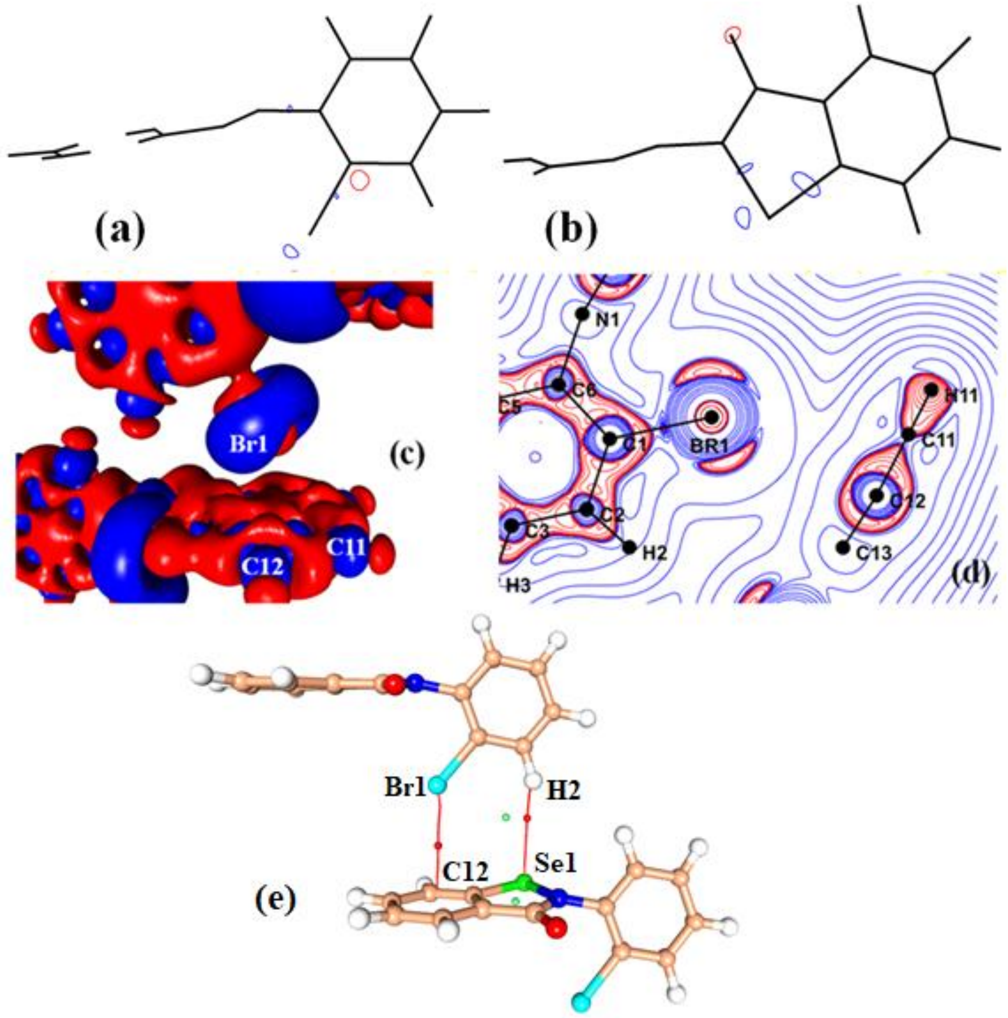

Figure S5 (a), (b): Residual density plots after theoretical multipolar refinement drew at 0.1 e/Å^3 contour level. (c), the 3D deformation density plot depicting the Br…π interaction region after theoretical multipolar refinement drawn at 0.05 e/Å^3 contour level. (d) 2D Laplacian plot depicting the Br…π interaction region after theoretical refinement drawn at logarithmic scale (e) Molecular graph depicting Br…C(π) and H…Se bcp (in blue) obtained from the theoretical model.

S2. Intermolecular Interactions

Table S1 List of important intermolecular interaction present in the crystal structure of **α-Se**

Interaction	Distance (Å)	∠ (°)	Symmetry Code
**N1-Se1···O1**	2.667(2)	174	0.5+x, 0.5-y, 0.5+z
**C12-H12···O1**	2.37	122	0.5+x, 0.5-y, 0.5+z
**C1-Br1···C12(π)**	3.304(2)	142	1+x, y, z
**C5-H5···O1**	2.47	136	1-x, 1-y, 1-z
**C3-H3···O1**	2.56	148	-x, 1-y, 1-z
**C3-H3···Se1**	2.83	153	0.5-x, 0.5+y, 1.5-z
**C10-H10···C3(π)**	2.80	144	0.5+x, 0.5-y, -0.5+z

S3. Topological Analysis

Table S2 Topological parameters for intramolecular bonds from experiment and theory (**Crystal09,** *in italics)*

**Bond**	**R_{ij} (Å)**	**ρ (e/Å^3)**	**$\nabla^2\rho$ (e/Å^5)**	**λ_1**	**λ_2**	**λ_3**	**ε**
**Se1-N1**	1.888	0.99	4.86	-4.47	-3.58	12.91	0.25
	1.881	*0.95*	*1.76*	*-4.67*	*-3.17*	*9.61*	*0.47*
**Se1-C13**	1.888	1.05	-0.30	-5.13	-4.09	8.92	0.25
	1.887	*0.89*	*0.91*	*-4.23*	*-2.51*	*7.66*	*0.69*
**Br1-C1**	1.888	0.96	1.32	-5.37	-4.07	10.76	0.32
	1.886	*0.94*	*0.70*	*-3.88*	*-3.45*	*8.02*	*0.12*
**O1-C7**	1.241	2.95	-28.30	-26.39	-24.03	22.13	0.10
	1.240	*2.64*	*-25.71*	*-22.84*	*-19.67*	*16.79*	*0.16*
**N1-C6**	1.418	2.00	-17.70	-14.30	-12.99	9.59	0.10
	1.415	*1.80*	*-10.83*	*-12.89*	*-11.98*	*14.05*	*0.07*
**N1-C7**	1.362	2.26	-27.50	-18.06	-14.12	4.67	0.28
	1.359	*2.22*	*-22.48*	*-17.37*	*-14.87*	*9.76*	*0.17*
**C8-C7**	1.471	1.96	-12.51	-14.46	-12.46	14.41	0.16
	1.466	*1.59*	*-9.75*	*-10.94*	*-9.23*	*10.41*	*0.18*
**C8-C13**	1.397	2.03	-16.67	-15.13	-12.00	10.46	0.26
	1.394	*1.96*	*-13.98*	*-13.62*	*-11.76*	*11.41*	*0.16*
**C8-C9**	1.393	2.19	-19.24	-17.29	-14.55	12.60	0.19

	1.398	*1.93*	*-14.29*	*-13.97*	*-11.57*	*11.25*	*0.21*
**C6-C5**	1.393	2.23	-20.75	-17.18	-13.96	10.39	0.23
	1.392	*2.04*	*-17.53*	*-15.45*	*-12.17*	*10.09*	*0.27*
**C6-C1**	1.393	2.07	-18.32	-16.30	-12.87	10.86	0.26
	1.395	*1.99*	*-15.75*	*-14.57*	*-11.63*	*10.45*	*0.25*
**C2-C1**	1.386	2.06	-18.27	-16.40	-13.64	11.77	0.20
	1.391	*2.08*	*-16.91*	*-14.74*	*-12.87*	*10.70*	*0.16*
**C2-C3**	1.388	2.11	-18.84	-16.47	-13.80	11.42	0.19
	1.389	*2.04*	*-17.55*	*-15.40*	*-12.58*	*10.43*	*0.22*
**C2-H2**	1.072	1.82	-16.82	-17.90	-17.38	18.46	0.03
	1.063	*1.84*	*-16.22*	*-17.37*	*-16.61*	*17.76*	*0.05*
**C12-C11**	1.390	2.12	-18.84	-16.50	-13.82	11.48	0.19
	1.387	*2.04*	*-17.58*	*-15.40*	*-12.59*	*10.41*	*0.22*
**C12-C13**	1.394	2.09	-19.66	-16.19	-13.43	9.96	0.21
	1.395	*2.04*	*-16.70*	*-15.08*	*-12.49*	*10.88*	*0.21*
**C12-H12**	1.075	1.81	-16.74	-17.87	-17.41	18.54	0.03
	1.063	1.84	-16.19	*-17.39*	*-16.54*	*17.74*	*0.05*
**C10-C11**	1.402	2.08	-18.18	-16.17	-13.67	11.66	0.18
	1.400	*2.03*	*-17.04*	*-15.27*	*-12.45*	*10.68*	*0.23*
**C10-C9**	1.385	2.12	-19.07	-16.54	-13.88	11.34	0.19
	1.386	*2.05*	*-17.78*	*-15.47*	*-12.63*	*10.32*	*0.22*
**C10-H10**	1.076	1.79	-16.23	-17.51	-17.11	18.39	0.02
	1.065	*1.82*	*-15.89*	*-17.26*	*-16.37*	*17.74*	*0.05*
**C4-C5**	1.390	2.11	-18.65	-16.36	-13.76	11.48	0.18
	1.390	*2.04*	*-17.47*	*-15.41*	*-12.56*	*10.49*	*0.23*
**C4-C3**	1.390	2.12	-18.94	-16.46	-13.94	11.46	0.18
	1.391	*2.05*	*-17.73*	*-15.56*	*-12.67*	*10.50*	*0.23*
**C4-H4**	1.074	1.80	-16.52	-17.59	-17.39	18.45	0.01
	1.066	*1.82*	*-15.93*	*-17.21*	*-16.48*	*17.75*	*0.04*
**C11-H11**	1.073	1.81	-16.77	-17.87	-17.32	18.43	0.03
	1.063	*1.83*	*-16.09*	*-17.32*	*-16.48*	*17.71*	*0.05*
**C5-H5**	1.073	1.81	-16.74	-17.87	-17.32	18.45	0.03
	1.065	*1.85*	*-16.23*	*-17.47*	*-16.62*	*17.87*	*0.05*
**C3-H3**	1.073	1.82	-16.84	-17.93	-17.33	18.43	0.03
	1.061	*1.84*	*-16.27*	*-17.42*	*-16.55*	*17.70*	*0.05*
**C9-H9**	1.072	1.81	-16.70	-17.79	-17.32	18.40	0.03
	1.062	*1.83*	*-16.23*	*-17.34*	*-16.55*	*17.65*	*0.05*

Table S3 Topological parameters for the various intermolecular interactions obtained from experiment and theory (*in italics*)

Intermolecular Interaction	R_{ij} (Å)	ρ (e/Å^3)	$\nabla^2\rho$ (e/Å^5)	ε	G (kJmol^{-1} bohr^{-3})	V (kJmol^{-1} bohr^{-3})	|V|/G
**C5-H5···O1**	2.578	0.06	0.91	0.82	19.52	-14.18	0.73
	2.517	*0.06*	*0.84*	*0.14*	*18.28*	*-13.62*	*0.74*
**C3-H3···O1**	2.615	0.04	0.68	0.10	13.57	-8.73	0.64
	2.595	*0.04*	*0.63*	*0.06*	*13.20*	*-9.16*	*0.69*
**Se1···O1**	2.657	0.18	2.14	0.28	56.94	-55.67	0.98
	2.667	*0.21*	*2.16*	*0.07*	*62.18*	*-65.52*	*1.05*
**C12-H12···O1**	2.446	0.07	1.17	0.09	25.44	-19.03	0.75
	2.427	*0.08*	*1.14*	*0.09*	*25.24*	*-19.52*	*0.77*
**C3-H3···Se1**	2.862	0.05	0.65	0.06	13.76	-9.84	0.71
	2.8350	*0.07*	*0.67*	*0.07*	*15.91*	*-13.67*	*0.86*
**C10-H10···C3(π)**	3.139	0.04	0.54	0.66	11.77	-7.71	0.65
	3.118	*0.04*	*0.47*	*0.76*	*8.89*	*-6.52*	*0.73*

S4. CSD Analysis

S4.1. Analysis of ebselen derivatives in CSD

In the ebselen derivative reported in the study (**α-Se**), the two phenyl rings (Cg1: C1-C6; Cg2: C8-C13) present in the molecule were perpendicular to each other in the crystal structure. The angle between the planes formed by these two rings was calculated to be 83.8° [Fig. S8(a)]. This deviation from planarity allows the Br to form a short C-Br···π interaction without any steric hindrance. Due to this important observation, it was important to analyses the angle between the planes formed by these two rings in the other ebselen derivatives present in the Cambridge Structural Database (CSD) [version 2017][24]. Our search, presented in Figure S7, resulted in a total of 16 structures. All molecules consisted of one molecule in the asymmetric unit except one (refcode: EDIGUU1) which had two molecules in the asymmetric unit. The CSD study shows that in most of the reported structures the molecular geometry is inclined towards planarity. However, in two of the structures, the inter-planar angle was calculated to

be 65.3° (EDIHAB) and 80.3° (QINYOD), respectively. This shows that the inter-planar angle of 83.8° observed in our molecule is the most nonplanar conformation of an ebselen derivative ever reported.

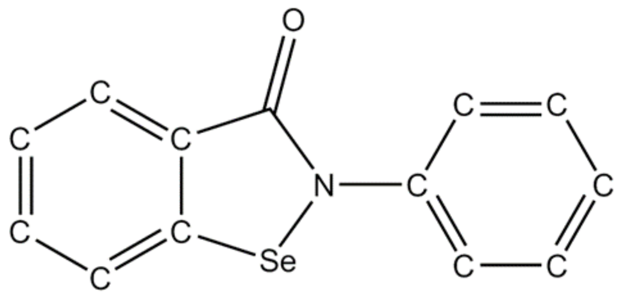

Figure S6 Search query as given in the CSD. Only structures whose 3D structure has been determined and whose R-factor < 0.1 were included in the search. In addition to that, ionic, disordered, polymeric and powder structures were removed from the search.

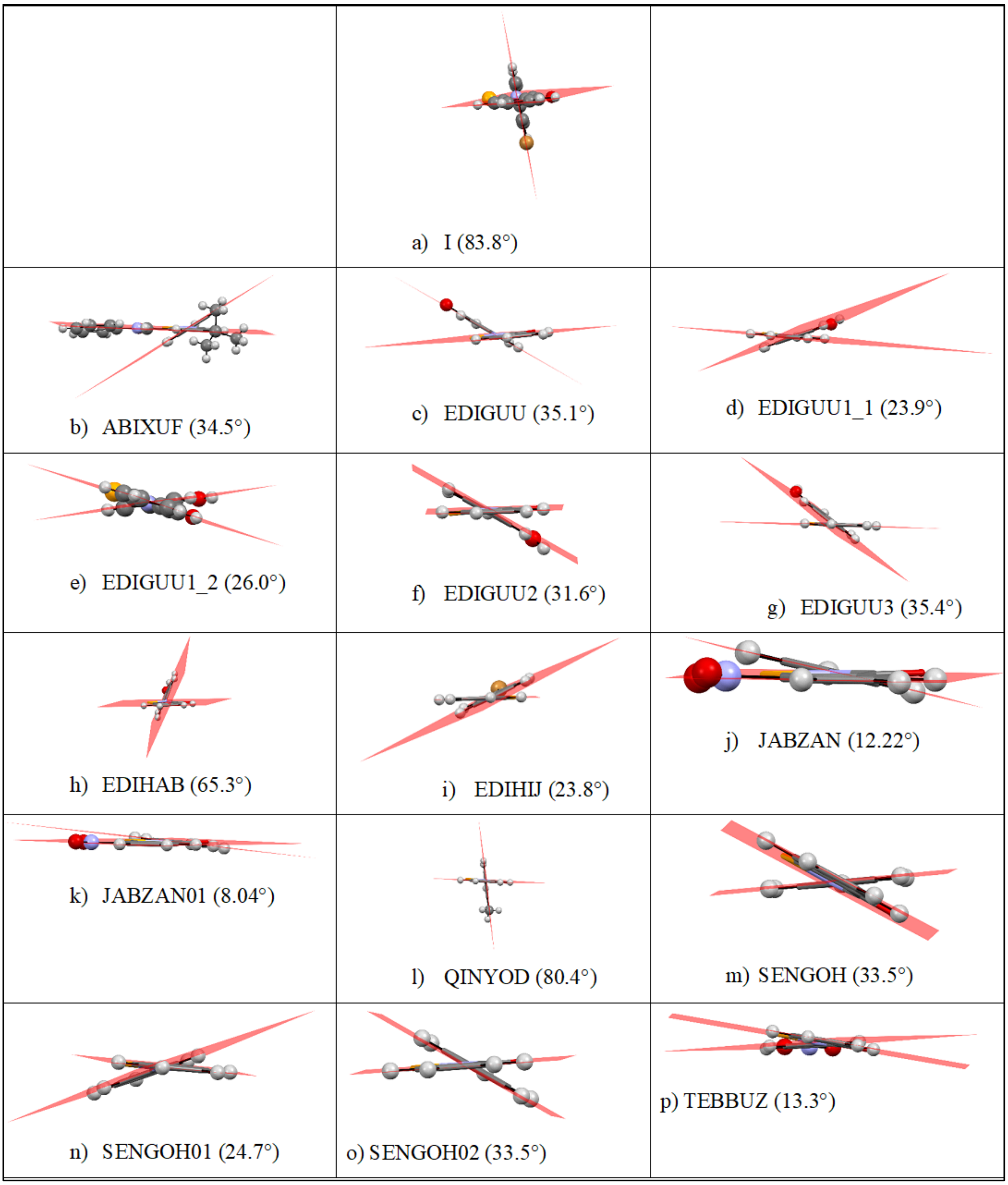

Figure S7 (a): Angle between Cg1/Cg2 planes in **α-Se;** (b)-(r): Angle between Cg1/Cg2 planes in structures retrieved from the CSD.

S4.2. C-Br···π interaction in the CSD

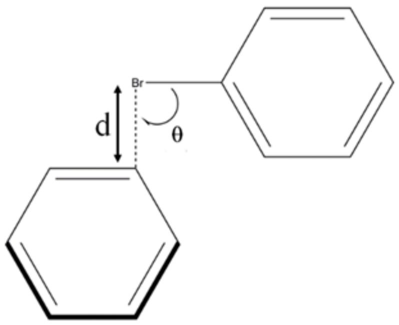

Figure S8 A search query for C-Br···C(π) interaction in the CSD. Only structures whose 3D structure has been determined and whose R-factor < 0.1 were included in the search. In addition to that, ionic, disordered, polymeric and powder structures were removed from the search.

Based on the C-Br···π(C) interaction described in our study, we investigated the presence of similar C-Br···π interaction in the CSD [Fig. S9]. First, we search for all C-Br···π(C) interactions with being less than the sum of the van der Waals (vdW) radius of Br and C atom. The angularity (∠ C-Br…C) ranged from 90° to 180°. This resulted in a total of 1467 hits having 2225 unique interactions. The mean distance and mean angle for all the interactions were calculated to be 3.46 Å and 154°, respectively [Fig. S10]. In the second step, we searched for structures with d ≤vdW (Br + C) – 0.2 Å and angularity ranged from 120° to 150°. in order to analyze the interactions closer to the geometry of the Br···π interaction in our crystal. This resulted in 21 hits having 22 unique interactions [Table S4]. Analysis of the geometrical parameters for these 22 interactions reveals the presence of only one interaction whose *d* was less than that reported in this study. This shows that the Br···π interaction reported in this study is one of the shortest interaction of its type.

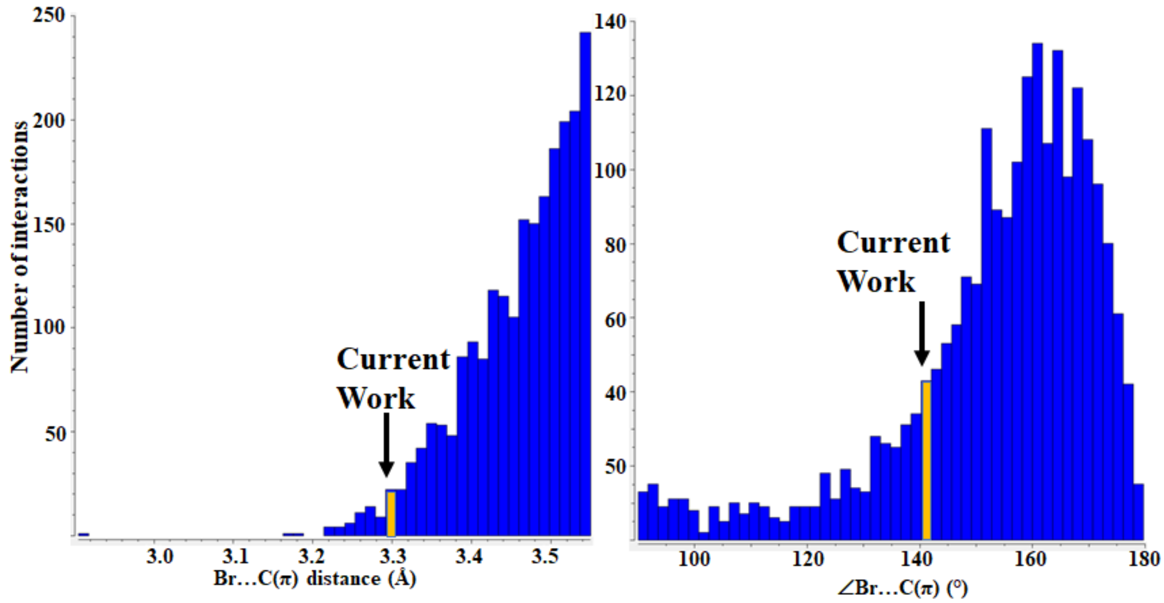

Figure S9 Distance and angularity distributions of Br···π(C) interaction present in the CSD.

Table S4 Refcodes and parameters of structures retrieved with d ≤vdW (Br + C) – 0.2 Å and 120° ≤∠ C-Br•••C(π) ≤ 150° in the CSD.

Refcode	Geometry (Å, °)	Refcode	Geometry (Å, °)
COQTOS	3.302/144	ROMCIH	3.311/ 144
ETINAW	3.333 / 150	SINFAY	3.322/ 149
IKEHUC	3.330/ 149	SUZTUB	3.322/ 148
IKUKUU	3.295/ 145	TAFVII	3.347/ 144
ISEDEQ	3.270/ 147	YIJCIF	3.349/ 149
LAZTIQ	3.332/ 149	YOBLOG	3.347/ 133
LIDMAN	3.313/ 145	ZACJIZ	3.342/ 132
LUFTUB	3.325/ 147 ; 3.344/ 148	ZUWWIZ	3.314/ 150
MONSIR01	2.905/ 139	EZUHUE	3.343/ 145
PATVUD	3.327/ 132	SOFVUG04	3.327/ 121
RAVDEY	3.341/ 143		
